# Cross-Cultural Adaptation and Validation of the Portuguese Version of the “Australian Pelvic Floor Questionnaire”

**DOI:** 10.1007/s00192-025-06087-0

**Published:** 2025-03-08

**Authors:** Marina Mesquita, Luís Cavalheiro, Pedro Ferreira, Rui Soles Gonçalves, Sónia Vicente

**Affiliations:** 1 Alcoitão School of Health Sciences, Cascais, Portugal; 2 Coimbra Health School, Polytechnic University of Coimbra, Coimbra, Portugal; 3 Center for Health Studies and Research of University of Coimbra (CEISUC), Coimbra, Portugal; 4 Faculty of Economics at the University of Coimbra, Center for Health Studies and Research of University of Coimbra, (CEISUC), Coimbra, Portugal; 5 Egas Moniz Center for Interdisciplinary Research (CiiEM), Caparica, Portugal; 6 Egas Moniz School of Health and Science, Caparica, Portugal

**Keywords:** Pelvic floor dysfunction, APFQ_P, Portuguese instrument, Women’s pelvic assessment instrument, Validation

## Abstract

**Introduction and Hypothesis:**

Pelvic floor dysfunction (PFD) is a common problem that occurs among women and increases with age and weight. This study was aimed at cross-culturally adapting and validating the original version of the Australian Pelvic Floor Questionnaire (APFQ) into Portuguese.

**Methods:**

The process of cultural and linguistic adaptation and validation followed the guidelines. The obtained Portuguese version was assessed by an expert panel of physiotherapists specialized in women’s health. Women with pelvic floor dysfunction also participated in a cognitive pre-test (*n* = 9). A sample of 50 women with PFD completed the questionnaire to evaluate internal consistency, construct validity, reproducibility, floor/ceiling effects assessment, and standard error of measurement. Test–retest was assessed with a 2-week interval. The study was approved by the Ethics Commission and all participants signed an informed consent form.

**Results:**

Fifty women with a mean age of 53.90 (± 18.57) years, BMI of 27.5 (± 4.2), 55.6% with a bachelor’s degree, and all with at least one child, participated in the study. The psychometric properties of the APFQ showed a high Cronbach’s alpha for the four domains: bladder (0.837), bowel and sexual function (0.756), pelvic organ prolapse (0.840), and total score (0.714). In terms of reproducibility, intraclass coefficient domain values ranged from 0.934 to 0.976, with a total score of 0.948.

**Conclusions:**

The APFQ was cultural and linguistically adapted and validated for Portuguese. The Portuguese version of the APFQ (APFQ_P) showed acceptable values of validity and good reliability. It can be used in both clinical evaluation and in research on pelvic floor dysfunction.

## Introduction

It is estimated that women have a 25% risk of developing pelvic floor dysfunction (PFD) [1]. According to the International Urogynecology Association and the International Continence Society, the definition of PFD includes 250 isolated manifestations of associated conditions, signs, and symptoms [2]. According to the literature, PFDs are associated with voiding and bowel dysfunction, pelvic organ prolapse, sexual dysfunction, and pelvic pain [3]. However, the three most common conditions are urinary and fecal incontinence and pelvic organ prolapse [2]. Many women experience one or more pelvic floor disorders throughout their lives [4]. Worldwide, the prevalence of PFD is rising in proportion to the global increase in aging and obesity [1], leading to a significant increase in public health problems associated with these conditions [4]. It is estimated that around 200 million people worldwide suffer from urinary incontinence [5]. In Portugal, according to the 2014 National Health Survey, it is estimated that more than 650,000 people over the age of 15 suffer from urinary incontinence, affecting mostly women (69.5%). These PFD symptoms are common, and although not life threatening, they affect quality of life [6]. The Women’s Preventive Services Initiative recommends annual PFD screening for all women, regardless of age or parity. Screening should assess whether women have symptoms and how they affect quality of life [1]. Therefore, several questionnaires assess quality of life and specific conditions of pelvic floor symptoms, as well as the negative impact on women’s daily lives [7]. Although these questionnaires are useful, especially in outcome research, most of them do not evaluate all domains of PFD: bladder, bowel, pelvic organ prolapse, and sexual symptoms. There are many instruments to assess incontinence and urinary obstruction, but fewer to assess pelvic organ prolapse and sexual symptoms, anal incontinence or anorectal obstruction [8]. Measurement tools can facilitate patient involvement in making decisions about their care and can help health professionals to identify patient concerns and outline goals for pelvic physiotherapy sessions [2]. However, when conducting a review of women’s health and pelvic floor instruments, the Australian Pelvic Floor Questionnaire (APFQ) can be used to screen for all PFDs [9]. The APFQ obtained acceptable Cronbach’s alpha coefficients across all domains, with the following values: bladder function (0.80), bowel function (0.73), sexual function (0.69), and pelvic organ prolapse (0.83). Despite the slightly lower alpha coefficient for the sexual function domain, it was not possible to remove items to improve the internal consistency without compromising the content validity of this domain. Regarding test–retest reliability, the Kappa coefficients ranged from 0.64 to 1.0, indicating substantial to almost perfect agreement across assessments [10]. This questionnaire has been translated and adapted for several countries, including China, Serbia, France, Turkey, Hungary, and Spain [6, 8, 11–14]. This study was aimed at cross-culturally adapting and validating the original version of the APFQ into European Portuguese.

## Materials and Methods

The original author was contacted to obtain permission for the study before starting the process of translating and validating the instrument. The study was conducted in accordance with the Declaration of Helsinki and it was approved by the Ethics Committee. The women’s participation was voluntary, the anonymity of the participants was guaranteed, and written informed consent was obtained from all women, after the nature and objectives of the study had been fully explained to them.

### Cross-Cultural Adaptation

1. The guidelines provided by the Center for Health Studies and Research, as well as the international recommendations outlined by Beaton et al. [15], were followed to culturally adapt the APFQ for use in European Portuguese translation: with the permission of the original author, two native translators independently translated the APFQ into Portuguese. These two versions were reviewed by an expert committee and by the author of the study. Discrepancies were discussed and debated until a consensus version was reached.
2. Back translation: the Portuguese consensus version was back-translated into English by another independent translator, the original and translated versions were compared, discrepancies discussed, and a pre-final version was produced.
3. Review: a committee of three physiotherapists with expertise in women’s health was set up to analyse cross-cultural equivalence between the original and Portuguese versions of the AFPQ. They provided a clinical review of the quality of the translation.
4. Pretest: a convenience sample of nine women with PFD participated in the cognitive pretest. They assessed the level of comprehension, the clarity of the language, the cultural relevance of the questionnaire, and the need to modify an item of the APFQ. An interview was conducted to understand if the women felt any difficulties in answering the items and to suggest solutions for a better formulation.

### Participants

Participants were recruited at a rehabilitation clinic, between June 2021 and April 2022. A sample of 50 women were eligible to participate in the study. They had been previously diagnosed with PFD, met the inclusion and exclusion criteria, and accepted the invitation to participate. The inclusion criteria of the study were women over 18 years of age, with full understanding of the spoken and written Portuguese language, with at least one symptom of PFD, urinary and\or fecal incontinence, pelvic organ prolapse, and sexual dysfunction and signed informed consent. Participants with cognitive and neurological disorders were excluded. Validity of the APFQ included assessment of reliability (internal consistency, reproducibility, and standard error of measurement), construct validity, and floor/ceiling effects.

The pretest sample (*n* = 9) was solely used to validate the clarity, appropriateness, and cultural relevance of the questionnaire, without integration into the main sample used for psychometric analysis (*n* = 50).

### Instruments

Each participant completed a sociodemographic characterization questionnaire, the Portuguese version of the APFQ, the Portuguese version of the Ditrovie Scale (10 items), and the Portuguese version of the EQ-5D-5L.

1. Sociodemographic and clinical questionnaire: information collected included age, education, occupation, weight, height, number of children and births, comorbidities, and previous pelvic surgery.
2. APFQ (Portuguese version): the APFQ assesses all aspects of PFD—bladder, bowel, pelvic organ prolapse and sexual symptoms. This questionnaire was developed for the “Longitudinal Assessment of Woman” study, conducted by the Betty Byrne Henderson Research Center, to collect longitudinal data on the incidence and prevalence of PFD in community-dwelling women [14]. The APFQ is an ordinal scale that assesses pelvic floor function and includes four domains: bladder function (questions 1 to 15), bowel function (questions 16 to 27), prolapse symptoms (questions 28 to 32), and sexual function (questions 33 to 42). To measure the frequency, severity, and discomfort of pelvic floor symptoms, responses are obtained using a Likert scale from least to most severe. The four-point scoring system is used for most items except for bowel frequency (question 16), bowel consistency (question 17), adequate lubrication (question 35), and reason for sexual abstinence (question 34). The APFQ consists of 42 multiple-choice questions. Except for the bowel function domain, there is an open-ended question at the end of each domain for other symptoms. Women can only mark one answer for each question, and scores are calculated separately for each domain. The calculated scores are divided by the number of questions in each domain and multiplied by 10. A value from 0 to 10 is obtained for each of the four domains and a total score of 40 points is obtained in the questionnaire [10].
3. Ditrovie Scale (10 items): the scale is designed to measure the psychological and functional impact of urinary incontinence on women’s health status and quality of life, and the impact of urinary incontinence on health care. It is self-administered and divided into five domains: activity, self-image, emotional impact, sleep, and general well-being. Dimension scores are displayed on a negative scale from 1 (best health status) to 5 (worst health status). It was validated in Portuguese with a Cronbach α of 0.93, good reproducibility (0.79), and construct validity with the analogic visual scale *r* = 0.72 for urinary impact, ranging from −0.391 to −0.572 with the 36 Medical Outcomes Study-short form (MOS-SF-36) [16].
4. EQ-5D-5L (Portuguese version): the EQ-5D-5L is a questionnaire for measuring health-related quality of life, which allows a value of an individual’s state of health to be generated. It consists of five domains: mobility, self-care, usual activities, pain/discomfort, and anxiety/depression. Each of these domains has five associated levels of severity, with level 1 corresponding to no problems, and level 5 corresponding to extreme problems. This system allows a total of 3125 different health states to be described. The estimated mean EQ-5D-5L index for Portugal’s general population is 0.887 (standard error [SE] = 0.0051), and the EQ VAS score was estimated as 76.0 (SE = 0.640) [17].

### Statistics

All data were analyzed using Microsoft Excel software and SPSS, version Statistics 28.0. Descriptive statistics was performed for sample analysis and Cronbach α was determined for internal consistency. Intraclass correlation coefficients with a 95% confidence interval was obtained for test–retest reliability. Spearman’s correlation coefficient was calculated to assess construction validity.

#### Reliability

Reliability was calculated and related to three concepts: internal consistency, reproducibility, and measurement error [16]. Cronbach’s α coefficient was used to calculate internal consistency. An acceptable value for Cronbach’s α is between 0.70 and 0.90 and represents a good internal consistency [17, 18]. Test–retest reproducibility or reliability was evaluated using the intraclass correlation coefficient (formula 2.1), which was used to measure and estimate the stability of continuous variables, as it takes into account measurement errors [18]. The questionnaires were repeated 2 weeks apart. No participant received treatment between these 2 weeks. Finally, standard error of measurement was calculated using the formula EPM = σ√1 – r, where σ corresponds to the standard deviation of the scores and r to the intraclass correlation coefficient [17].

#### Validity

To assess the construct validity of the Portuguese version of the APFQ, Spearman’s correlation coefficients were calculated between the scores of this instrument and the Ditrovie Scale (10 items), the EQ-5D-5L, and the main sociodemographic data. Correlation coefficients should be interpreted as follows: very high if greater than 0.90; high if between 0.70 and 0.89; moderate if between 0.40 and 0.69; low if between 0.20 and 0.39; and very low if less than or equal to 0.19 [19].

In this study, six predefined hypotheses were also tested to assess construct validity:

- Hypothesis 1: we expected to obtain high correlation values between the different domains of the Ditrovie Scale (10 items) and bladder function from the APFQ.
- Hypothesis 2: we expected to obtain no correlation values, or very weak correlation values, between the domains of the Ditrovie Scale (10 items) and bowel function, pelvic organ prolapse, and sexual function from the APFQ.
- Hypothesis 3: we expected to obtain moderate correlation values between the different domains of the Ditrovie Scale (10 items) and the APFQ total score.
- Hypothesis 4: we expected to obtain moderate correlation values between the EQ-5D-5L scores and the different domains of the APFQ.
- Hypothesis 5: we expected to obtain moderate correlation values between age and body mass index and the dimensions of the APFQ.
- Hypothesis 6: we expected to obtain statistically significant differences in favor of practicing physical activity in the different dimensions of the APFQ.

## Results

For cross-cultural adaptation, the results of the clinical review of the quality of the translation were obtained by the three experts, who concluded that the translation was generally faithful to the original questionnaire and appropriate for the Portuguese population. A sample of women (*n* = 9) with pelvic floor dysfunction participated in the pretest. The mean age of the participants was 50 (± 5.4) years, the mean BMI was 27.5 (± 4.2), the majority had at least a bachelor’s degree, their occupations were diverse, all had at least one child, and the majority had two children (66.7%). The most common forms of PFD were stress urinary incontinence (33.3%), fecal incontinence (22.3%), and mixed urinary incontinence (44.4%). Participants took an average of 6.7 ± 3 min, ranging from 3.5 to 12 min, to complete the questionnaire. The components evaluated during the pretest included the participants’ understanding of the translated questionnaire, the time taken to complete it, and their feedback on the clarity and relevance of the questions. In the follow-up interview, which lasted an average of 15 min, participants reported that the questionnaire was brief, easy to answer, understandable, and suitable for the population with PFD. Minor difficulties were pointed out with specific terminology, leading to slight revisions for improved clarity. Regarding the pretest, it was concluded that no doubts in answering the instrument were indicated; thus, the research group accepted the final Portuguese version of the self-administered APFQ.

A convenience sample of 50 women participated in the psychometric properties study. The mean age was 54 (± 18.57) years, 62% were married, 56% had higher education, and there were a variety of occupations. Regarding physical activity (Table 1), 54% of women did some type of activity for more than 2 h. Most were nonsmokers, 36% had two children, 50% had had a vaginal delivery, and 62% had had no episiotomy.

**Table 1:** Characteristics of the pretest sample

	Mean ± standard deviation (minimum to maximum)	*n*	Percentage
Age (years)	50 ± 15.4 (27–75)	9	
BMI	27.5 ± 4.2 (20–34.7)	9	
Level of education			
≤ High school		4	44.4
≥ Bachelor		5	55.6
Occupation			
Nurse		1	11.1
Teacher		1	11.1
Domestic		1	11.1
Head of Department at the Ministry of Education		1	11.1
Jurist		1	11.1
Psychologist		1	11.1
Administrative		1	11.1
Attorney		1	11.1
Marketeer		1	11.1
With children		9	100
Number of children			
1 Child		3	33.3
2 Children		6	66.7
Pathology			
Stress urinary incontinence		3	33.3
Fecal incontinence		2	22.2
Mixed urinary incontinence		4	44.4

### Reliability: Internal Consistency

Cronbach’s α coefficient presented good values for all the domains, with 0.837 to bladder function, 0.756 bowel function, 0.840 pelvic organ prolapse, and 0.756 sexual function. Total score value of Cronbach’s α coefficient was 0.714 (Table 2).

**Table 2:** Characteristics of the psychometric sample

	*n*	Minimum	Maximum	Average	Standard deviation	Percentage
Age (years)	50	26	84	53.90	18.57	
Weight (kg)	50	50	105	64.98	10.93	
Height (m)	50	1.49	1.83	1.62	0.07	
BMI (kg\m2)	50	18.38	36.33	24.72	3.79	
Physical activity						
No	23					46
Yes	27					54
How many hours						
Didn’t respond	18					
Less than 1 h	6					
1 h	6					
2 h	7					
More than 2 h	13					
Total score	32					

*BMI* Body Mass Index

### Reproducibility

The intraclass coefficients (ICC; 95% confidence intervals) obtained were all very high according to the scale and varied between 0.934 and 0.976 in the different domains. The total score was 0.948 (Table 2).

### Standard Error of Measurement

Standard error measurement values in the different domains ranged from 0.20 to 0.36. The total score was 0.15.

### Floor/Ceiling Effects

The floor effects of the APFQ domains were found to be 78% and 32% for pelvic organ prolapse and sexual function respectively. The ceiling effect was zero for all domains.

### Construct Validity

High correlation values were obtained between the APFQ Bladder Function dimension and the Ditrovie Scale (10 item) domains (rho values between 0.716 and 0.726), as well as with the Ditrovie Scale (10 items) total score (rho = 0.782). Low to moderate correlation values were also observed between the APFQ total score and the Ditrovie Scale (10 item) domains (rho between 0.342 and 0.482). Only low and negative correlation values were observed between the APFQ total score and the EQ-5D-5L index and VAS scores respectively (−0.302 and −0.303 respectively; Table 3). Regarding the relationship between the variables age and BMI with the different domains of the APFQ, only low correlation values were found between BMI and bladder function (rho = 0.290) and low and negative correlation values between sexual function with age (rho = −0.324) and BMI (rho = −0.386). When comparing physical activity practice with the APFQ domains, statistically significant differences were observed only for the bladder-function dimension (rho = 0.027), which was unfavorable for physical activity practice.

**Table 3:** Internal consistency, standard error of measurement, floor/ceiling effect, and retest test

	Cronbach α	SEm	FE%	CE%	ICC	Confidence interval 95%	
						Lower limit	Upper limit
Bladder function	0.837	0.20	6.0	0.0	0.976	0.959	0.987
Function of the intestines	0.756	0.36	4.0	0.0	0.934	0.883	0.962
Pelvic organ prolapse	0.840	0.20	78.0	0.0	0.966	0.940	0.981
Sexual function	0.756	0.27	32.0	0.0	0.969	0.946	0.982
Total score	0.714	0.15	0.0	0.0	0.948	0.908	0.970

*SEm* standard error of measurement, *FE%* floor effect, *CE%* ceiling effect, *ICC* intraclass correlation coefficient

The average scores obtained by the APFQ sample are very low, ranging from 0.47 (± 1.18) on symptoms of prolapse to 1.97 (± 1.40) in bladder function. Function of the intestines was 1.98 (± 1.37), sexual function was 1.30 (± 1.54), and total score was 1.41 (± 0.66). These results may indicate a small impact on the health status of the participants (Table 4).

**Table 4:** Construct validity of APFQ domains vs Ditrovie Scale (10 items) and EQ-5D-5L

		Activities of daily living	Image	Emotional impact	Ditrovie global score	EQ-5D-5L	EQ-5D VAS
Bladder function	rho	0.726**	0.721**	0.716**	0.782**	−0.274	−0.030
	*p*	0.000	0.000	0.000	0.000	0.054	0.834
	*n*	50	50	50	50	50	50
Function of the intestines	rho	−0.142	0.056	−0.113	−0.003	−0.262	−0.246
	*p*	0.325	0.699	0.435	0.985	0.066	0.085
	*n*	50	50	50	50	50	50
Pelvic organ prolapse	rho	0.265	0.158	0.164	0.265	−0.154	−0.108
	*p*	0.062	0.272	0.255	0.063	0.287	0.456
	*n*	50	50	50	50	50	50
Sexual function	rho	0.089	0.013	0.007	0.073	0.045	0.054
	*p*	0.539	0.928	0.960	0.616	0.757	0.712
	*n*	50	50	50	50	50	50
Total score	rho	0.363**	0.417**	0.342*	0.482**	−0.302*	−0.303*
	*p*	0.010	0.003	0.015	0.000	0.033	0.032
	*n*	50	50	50	50	50	50

^*^The correlation is significant at the 0.05 level

^**^The correlation is significant at the 0.01 level

## Discussion

### Reliability: Internal Consistency

The translation and validation of the APFQ into European Portuguese were successfully conducted, providing a useful tool for clinical and research applications. Cronbach’s α coefficient values for the Portuguese version indicated good internal consistency for bladder function (0.837), bowel function (0.756), pelvic organ prolapse (0.840), and sexual function (0.756). These results are comparable with the original version, which reported values of 0.80 for bladder function, 0.73 for bowel function, 0.83 for pelvic organ prolapse, and 0.69 for sexual function [10]. The slightly higher values in the translated version for bladder, bowel, and pelvic organ prolapse domains reflect consistent performance across languages, whereas the sexual function domain maintained an acceptable level of reliability.

### Reproducibility

Reproducibility was supported by ICCs for bladder function (0.976), bowel function (0.943), pelvic organ prolapse (0.966), and sexual function (0.969), showing strong test–retest reliability. The standard error of measurement (ranging from 0.20 to 0.36) was low, which may be attributed to the small sample size.

### Construct Validity

Construct validity was analyzed using the Ditrovie Scale (10 items), the EQ-5D-5L, and sociodemographic variables (age, BMI, and physical activity). Six pre-defined hypotheses based on the literature were partially confirmed, notably, a strong positive correlation (rho = 0.782) between the Ditrovie Scale (10 items) and the APFQ bladder function domain confirming hypothesis 1 and that these instruments measure similar constructs. As expected, the absence of correlations between the Ditrovie Scale and other APFQ domains, such as bowel function and sexual function, was observed, as they assess unrelated constructs and was consistent with hypothesis 2.

### Hypothesis Testing

Hypothesis 3 revealed a moderate correlation (rho = −0.482) between the total APFQ score and the Ditrovie Scale (10 items), attributed to the inclusion of bladder function items in the APFQ’s total score. Hypothesis 4 found correlations only with the total score of the EQ-5D-5L, which aligns with the APFQ’s negative orientation, where lower scores reflect better conditions.

The absence of expected correlations with other APFQ dimensions could be due to the sample size (*N* = 50) or the low severity of the conditions reported by participants (mean total score 1.41 with a standard deviation of 0. 66). The low average scores from the APFQ (bladder function 1.97 [SD = 1.40], function of the intestines 1.89 [SD = 1.37], symptoms of prolapse 0.47 [SD = 1.18], and sexual function 1.30 [SD = 1.54]) suggest a limited health impact on participants.

### Relationship with BMI and Physical Activity

In hypothesis 5, a low correlation between BMI and bladder function was noted, indicating that women with higher BMI scored worse in the APFQ. This aligns with findings from Milsom and Gyhagen [20], highlighting the role of obesity in developing urinary incontinence. Additionally, a negative correlation was observed between BMI and sexual function, suggesting that younger women with lower BMI had better scores, consistent with Peixoto and Nobre’s [21] findings on age-related sexual health issues.

The lack of correlations between BMI, age, and the other APFQ dimensions may be due to the sample characteristics or the comprehensive nature of the APFQ. The tool captures data across various conditions (urinary incontinence, fecal incontinence, pelvic organ prolapse, and sexual function), and the sample may not uniformly represent these issues, possibly focusing more on urinary incontinence. The original authors describe the APFQ as more suited for epidemiological studies, which may explain these results [14] Table 5.

**Table 5:** Construction validity of Australian Pelvic Floor Questionnaire domains with age and Body Mass Index

		Age	BMI
Bladder function	rho	0.097	0.290*
	*p*	0.502	0.041
	*n*	50	50
Function of the intestines	rho	0.046	0.030
	*p*	0.751	0.836
	*n*	50	50
Pelvic organ prolapse	rho	0.034	0.023
	*p*	0.813	0.876
	*n*	50	50
Sexual function	rho	−0.324*	−0.386**
	*p*	0.022	0.006
	*n*	50	50
Total score	rho	−0.031	−0.104
	*p*	0.831	0.472
	*n*	50	50

*The correlation is significant at the 0.05 level

**The correlation is significant at the 0.01 level

### Physical Activity and Bladder Function

Contrary to initial expectations, hypothesis 6 showed statistically significant differences between physical activity and bladder function (rho = 0.027). Women engaging in physical activity had worse scores in the bladder function domain. This may be explained by the stress placed on the pelvic floor during exercise, which can affect sphincter control [22]. Studies have also indicated a higher prevalence of stress urinary incontinence among female athletes [23] Table 6.

**Table 6:** Construction validity of the Australian Pelvic Floor Questionnaire and physical activity domains

	Physical activity	*n*	Average	Standard deviation	rho
Bladder function	No	23	2.46	1.65	
	Yes	27	1.56	1.00	0.027
Function of the intestines	No	23	1.97	1.64	
	Yes	27	1.82	1.12	0.704
Pelvic organ prolapse	No	23	0.41	1.04	
	Yes	27	0.52	1.31	0.741
Sexual function	No	23	1.47	1.49	
	Yes	27	1.16	1.59	0.488
Total score	No	23	1.58	0.70	
	Yes	27	1.26	0.60	0.093

### Floor/Ceiling Effects

The floor/ceiling effects analyzed in this study were notable in the pelvic organ prolapse domain (78% floor effect) and sexual function domain (32% floor effect), indicating high scores that could limit the detection of treatment improvements upon subsequent responses.

### Limitations

The study’s limitations include the small sample size and the low severity of conditions among participants, potentially affecting the generalizability of the findings. Additionally, the comprehensive nature of the APFQ may have led to a focus on a specific condition (e.g., urinary incontinence) rather than a balanced representation across all assessed domains.

Future studies should include larger and more diverse samples to enhance statistical power and ensure broader applicability of the results. Additionally, the relatively low severity of reported conditions might have influenced some correlations, suggesting the need for further investigation across different severity levels of pelvic floor dysfunction.

## Conclusion

The Portuguese version of the APFQ (APFQ_P) demonstrated strong psychometric properties in terms of reliability and validity. This validated version is a valuable tool for the comprehensive assessment of PFD and for decision-making by both health professionals and users. Data obtained from the APFQ, when combined with other clinical evaluation measures, can significantly contribute to research advancements and improved health care outcomes.

## Data Availability

The dataset used during the current study is published in open acess on mendeley Data and available from https://data.mendeley.com/datasets/yd4r53t3n2/1.
